# Assessment of Perceptions of the Public Charge Rule Among Low-Income Adults in Texas

**DOI:** 10.1001/jamanetworkopen.2020.10391

**Published:** 2020-07-15

**Authors:** Benjamin D. Sommers, Heidi Allen, Aditi Bhanja, Robert J. Blendon, E. John Orav, Arnold M. Epstein

**Affiliations:** 1 Department of Health Policy and Management, Harvard T.H. Chan School of Public Health, Boston, Massachusetts; 2 Department of Medicine, Brigham & Women’s Hospital, Boston, Massachusetts; 3 Columbia University School of Social Work, New York, New York

## Abstract

This survey study investigates perceptions of the Trump administration’s new public charge rule among low-income Texan adults.

## Introduction

In January 2020, the US Supreme Court allowed the Trump administration to implement a new rule regarding the definition of *public charge*. The rule establishes participation in public programs (Medicaid, the Supplemental Nutritional Assistance Program [SNAP], and subsidized housing), health status, and income as criteria in determining whether legal immigrants are able to gain permanent residency (green cards). Critics contend that this creates a chilling effect that dissuades individuals from participating in programs or obtaining medical care, even among people outside the policy’s intended scope (such as citizens with immigrant relatives).^1^ We surveyed low-income adults in Texas, a state with a large immigrant population, to examine perceptions of the public charge rule and its association with program participation and receipt of medical care.

## Methods

We conducted a random-digit dialing telephone survey in November and December 2019 among US citizens aged 19 to 64 years in Texas with family incomes below 138% of the federal poverty level. Our sample was limited to citizens for 2 reasons: this was part of a larger longitudinal study of health insurance focused on citizens using a validated method,^2^ and noncitizens may be reluctant to participate in this type of assessment, which could generate nonresponse bias not easily addressed through reweighting. The survey was available in English and Spanish and was administered on cellular and landline telephones. As an anonymous survey, the study was deemed exempt by the Harvard T.H. Chan School of Public Health’s institutional review board. Participants provided oral informed consent.

We used demographic weighting to reduce potential nonresponse bias.^3^ The survey assessed demographic characteristics, awareness of the public charge rule, and whether respondents had friends or family who had stopped participating or avoided public programs because they were “worried it would disqualify them from obtaining a green card or becoming a US citizen,” or who had avoided visiting a physician or a hospital “because they did not want to be asked about their immigration status.”

We calculated descriptive proportions and conducted logistic regression analysis for selected outcomes. Statistical significance for the logistic regression model was based on 2-tailed *P* value of .05. Data analysis was performed using Stata statistical software version 14.0 (StataCorp). Analyses were performed from January to February 2020.

## Results

The survey was completed by 400 adults (mean [SD] age, 38.6 [13.7] years), with a response rate of 7%, using the American Association for Public Opinion Research’s RR3 definition. The sampling frame corresponded to 3.9 million low-income citizens in Texas. The sample was 192 Latino respondents, (40.3%), 125 white respondents (35.5%), and 54 black respondents (17.5%); 213 female respondents (56.8%); and 84 rural respondents (13.6%) (Table); total unweighted numbers of respondents and weighted percentages are shown.

**Table.** Factors Associated With Awareness of the Public Charge Rule and Program Participation and Receipt of Medical Care^a^

Factor	Respondents (N = 400)		Heard of public charge rule (n = 396)		Knows friends or family who avoided or stopped participating in Medicaid, SNAP, or public housing (n = 397)		Knows friends or family who avoided visiting a physician, a clinic, or a hospital (n = 397)	
	Unweighted No.	Weighted %	Predicted probability, % (95% CI)	*P* value	Predicted probability, % (95% CI)	*P* value	Predicted probability, % (95% CI)	*P* value
Sex								
Male	187	43.2	63.0 (53.5 to 72.6)	.31	5.2 (1.8 to 8.6)	.63	11.2 (3.5 to 18.9)	.21
Female	213	56.8	56.3 (48.0 to 64.5)	[Reference]	6.5 (2.7 to 10.2)	[Reference]	5.9 (1.9 to 10.0)	[Reference]
Age, y								
19-30	105	36.9	41.1 (29.9 to 52.3)	<.001	3.8 (0.7 to 6.8)	.12	5.7 (0.9 to 10.5)	.54
31-45	111	29.9	60.9 (48.5 to 73.3)	.04	6.6 (1.1 to 12.1)	.63	10.7 (1.7 to 19.8)	.71
46-64	184	33.2	76.9 (68.2 to 85.7)	[Reference]	8.5 (3.3 to 13.7)	[Reference]	8.4 (1.1 to 15.7)	[Reference]
Race/ethnicity								
Latino	192	40.3	61.7 (51.8 to 71.5)	.92	10.1 (4.6 to 15.5)	<.001	11.8 (4.7 to 18.9)	.11
Not Latino								
White	125	35.5	62.4 (52.2 to 72.7)	[Reference]	0.8 (−0.1 to 1.7)	[Reference]	5.2 (0.4 to 10.0)	[Reference]
Black	54	17.5	53.5 (37.1 to 69.8)	.37	9.8 (0.7 to 19.0)	.001	7.3 (−3.7 to 18.3)	.71
Other	29	6.7	41.8 (18.8 to 64.9)	.12	NA^b^	NA	NA^b^	NA
Education								
Less than high school	93	21.0	57.8 (43.5 to 72.1)	.58	3.5 (0.1 to 7.0)	.09	4.7 (−2.4 to 11.9)	.26
High school graduate or equivalent	134	32.6	54.7 (44.8 to 64.5)	.26	4.7 (1.6 to 7.9)	.15	5.4 (1 to 9.7)	.10
Some college or college degree	173	46.3	62.8 (53.1 to 72.6)	[Reference]	8.9 (3.6 to 14.2)	[Reference]	12.2 (4.4 to 20.0)	[Reference]
Residence								
Urban or suburban	316	86.4	61.3 (54.6 to 68)	[Reference]	6.6 (3.6 to 9.5)	[Reference]	8.5 (4.1 to 12.8)	[Reference]
Rural	84	13.6	45.4 (31.3 to 59.5)	.05	1.3 (−0.8 to 3.4)	.04	5.2 (−0.8 to 11.2)	.44
Employment								
Not working	215	50.1	62.6 (53.9 to 71.4)	.30	4.9 (1.6 to 8.1)	.35	8.4 (2.3 to 14.5)	.86
Working	185	49.9	55.9 (46.8 to 64.9)	[Reference]	7.2 (3.3 to 11.2)	[Reference]	7.7 (2.5 to 12.9)	[Reference]
Chronic condition^c^								
None	139	34.7	68.0 (58.0 to 78)	[Reference]	4.5 (0.9 to 8.1)	[Reference]	5.4 (0.8 to 10.1)	[Reference]
≥1	261	65.3	54.0 (45.9 to 62.2)	.05	6.8 (3.1 to 10.5)	.40	9.7 (4.2 to 15.3)	.25

Abbreviations: NA, not applicable; SNAP, Supplemental Nutritional Assistance Program.

^a^ Data are from a telephone survey of 400 low-income Texans, who were all US citizens aged 19 to 64 years, with family incomes below 138% of the federal poverty level, conducted in November and December 2019. Sample size listed for each question excluded individuals who did not provide a response for that question. All analyses are from multivariate logistic regression models, with probabilities generated using the *margins* command in Stata statistical software version 14.0 (StataCorp), which uses the regression coefficients and the sample’s actual distribution of covariates. All estimates were survey-weighted to match the population features of the target population.

^b^ No one in the Other group (which included 11 people reporting multiple races, 5 Asian respondents, 4 Native American respondents, and 9 who did not report any race) experienced either of these outcomes, so this category was combined with white respondents for these models. Alternatively, excluding these 29 observations from these 2 models produces similar overall results.

^c^ Chronic conditions were the self-reported presence of any of the following conditions: hypertension, heart attack or coronary artery disease, stroke, asthma or chronic obstructive pulmonary disease, kidney disease, diabetes, depression, cancer, and substance abuse.

The survey found that 58.9% of respondents had heard of the public charge rule (Figure), mostly from news sources (50.2%) or social media (28.7%); only 0.8% had primarily heard from a physician’s office or hospital. Of those familiar with the rule, 45.2% were very concerned or somewhat concerned about its potential impact on their friends or families.

**Figure.**
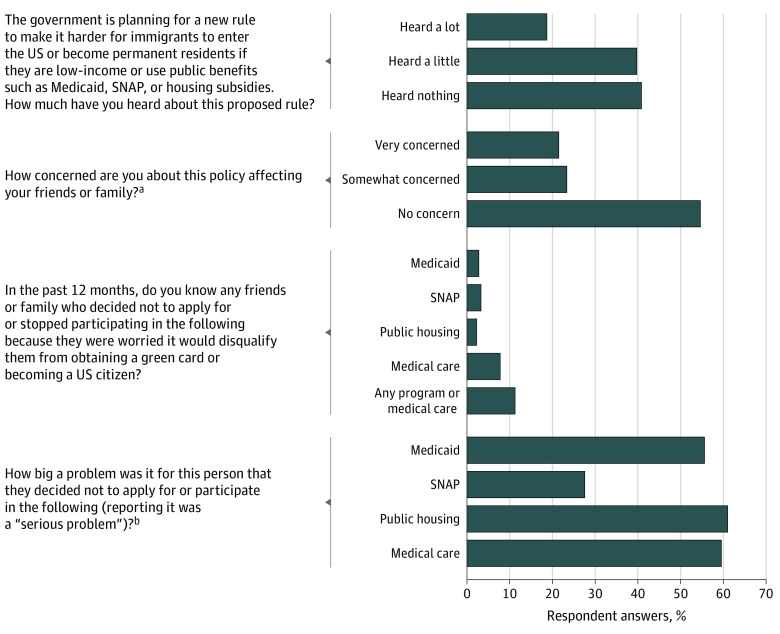
Perceptions of and Outcomes Associated With the Public Charge Rule Among Low-Income Adults in Texas Data are from a telephone survey of 400 low-income Texans, who were all US citizens aged 19 to 64 years, with family incomes below 138% of the federal poverty level, conducted in November and December 2019. SNAP indicates Supplemental Nutritional Assistance Program. ^a^This question was asked only of those who reported having heard about the public charge rule. ^b^This question was asked only of those who reported knowing someone avoiding program participation or obtaining medical care.

The survey also found that 11.6% of respondents (corresponding to over 400 000 low-income citizens statewide) reported knowing friends or family who had avoided participating in Medicaid, SNAP, or public housing, or had not visited a physician or hospital because of immigration-related concerns in the past year. Eight percent had avoided medical care, with smaller numbers avoiding Medicaid (2.9%), SNAP (3.5%), and public housing (2.4%). For all but SNAP participation, the majority said these changes were a serious problem.

Predicted probabilities for the awareness of the rule were lower among young adults aged 19 to 30 years (41.1% [95% CI, 29.9% to 52.3%] vs 76.9% [95% CI, 68.2% to 85.7%] among respondents aged 46 to 64 years; *P* < .001), rural residents (45.4% [95% CI, 31.3% to 59.5%] vs 61.3% [95% CI, 54.6% to 68.0%] among urban residents, *P* = .05), and people with chronic conditions (54.0% [95% CI, 45.9% to 62.2%] vs 68.0% [95% CI, 58.0% to 78.0%] among people with no conditions; *P* = .05) (Table). Avoidance of public programs was more commonly reported among Latino respondents (10.1% [95% CI, 4.6% to 15.5%] vs 0.8% [95% CI, −0.1 % to 1.7%] among white respondents; *P* < .001), black respondents (9.8% [95% CI, 0.7% to 19.0%] vs 0.8% [95% CI, −0.1 % to 1.7%] among white respondents; *P* = .001), and urban populations (6.6% [95% CI, 3.6% to 9.5%] vs 1.3% [95% CI, −0.8% to 3.4%] among rural residents; *P* = .04). There were no factors significantly associated with avoiding needed medical care.

## Discussion

In this timely survey, 11.6%, or nearly 1 in 8, low-income Texans had friends or family who avoided public programs or medical care in the past year because of immigration-related concerns. This represents a substantial population-level impact of nearly half a million people in Texas. This is consistent with reported reductions in Texas’s Medicaid enrollment linked to immigration concerns^4^ and national estimates that millions of legal immigrants may forego health insurance because of the public charge rule.^1,5^ These results are even more concerning in the context of the current coronavirus disease 2019 pandemic.

Study limitations include the low response rate, similar to other telephone polls.^6^ Reluctance to answer immigration-related questions—potentially associated with rising anti-immigrant sentiment nationwide—likely led us to underestimate the policy’s impact. Our sample came from a single state, although one with a sizeable immigrant population. Finally, the sample was limited to citizens asked about the experiences of friends and families; again, this likely led us to underestimate the changes associated with this policy in immigrant communities.
